# Host antimicrobial peptide S100A12 disrupts the fungal membrane by direct binding and inhibits growth and biofilm formation of *Fusarium* species

**DOI:** 10.1016/j.jbc.2024.105701

**Published:** 2024-01-30

**Authors:** Sanhita Roy, Bharathi Bhogapurapu, Sreyanki Chandra, Karishma Biswas, Priyasha Mishra, Abhijit Ghosh, Anirban Bhunia

**Affiliations:** 1 Prof. Brien Holden Eye Research Centre, LV Prasad Eye Institute, Hyderabad, India; 2 Dr. Chigurupati Nageswara Rao Ocular Pharmacology Research Centre, LV Prasad Eye Institute, Hyderabad, India; 3 Department of Chemical Sciences, Bose Institute, Unified Academic Campus, Kolkata, India; 4 Graduate Studies, Manipal Academy of Higher Education, Manipal, India

**Keywords:** S100A12, antimicrobial peptide, *Fusarium*, reactive oxygen species, biofilm

## Abstract

Fungal keratitis is the foremost cause of corneal infections worldwide, of which *Fusariumspp.* is the common etiological agent that causes loss of vision and warrants surgical intervention. An increase in resistance to the available drugs along with severe side effects of the existing antifungals demands for new effective antimycotics. Here, we demonstrate that antimicrobial peptide S100A12 directly binds to the phospholipids of the fungal membrane, disrupts the structural integrity, and induces generation of reactive oxygen species in fungus. In addition, it inhibits biofilm formation by *Fusariumspp.* and exhibits antifungal property against *Fusariumspp.* both *in vitro* and *in vivo*. Taken together, our results delve into specific effect of S100A12 against *Fusariumspp.* with an aim to investigate new antifungal compounds to combat fungal keratitis.

Fungal keratitis (FK) is a major cause of blindness in India and globally (1); often treatment is challenging leading to corneal transplantation. *Fusarium* *spp.* are the main etiological agents of FK worldwide and find its place in the fungal priority pathogen list recently released by the World Health Organization. It is also responsible for invasive infections of airways and skin. The escalating crisis of resistance toward existing antifungals repeatedly makes the situation worse. Currently available antifungal drugs have serious limitations, including poor efficacy, high toxicity, and low bioavailability (2). In addition, antifungal drug resistance, especially to azoles, is prevalent among immunocompromised individuals, and amphotericin often induces toxicity in these individuals (3). Therefore, development of novel antimicrobial agents with different mode of action, low toxicity, and high efficacy becomes essential. In this context, antimicrobial peptides (AMPs), natural or synthetic, are considered as potent alternative therapeutics. Human S100A12, a host defense peptide, consists of two EF hand domains, forms homo-oligomers, and binds various transition metal ions, including Zn^2+^ and Ca^2+^. Others and our previous studies revealed its antibacterial activity against several pathogens including *Helicobacter pylori* (4), *Campylobacter jejuni* (5), and *Pseudomonas aeruginosa* (6). Here, we examined the antifungal efficiency of S100A12 against filamentous *Fusarium* *spp.* along with its mechanism of action, with an aim to develop a potent antifungal drug for combating fungal infections.

## Results

### S100A12 inhibits growth and biofilm formation of *Fusariumspp.* and induces generation of reactive oxygen species

**Figure 1:**
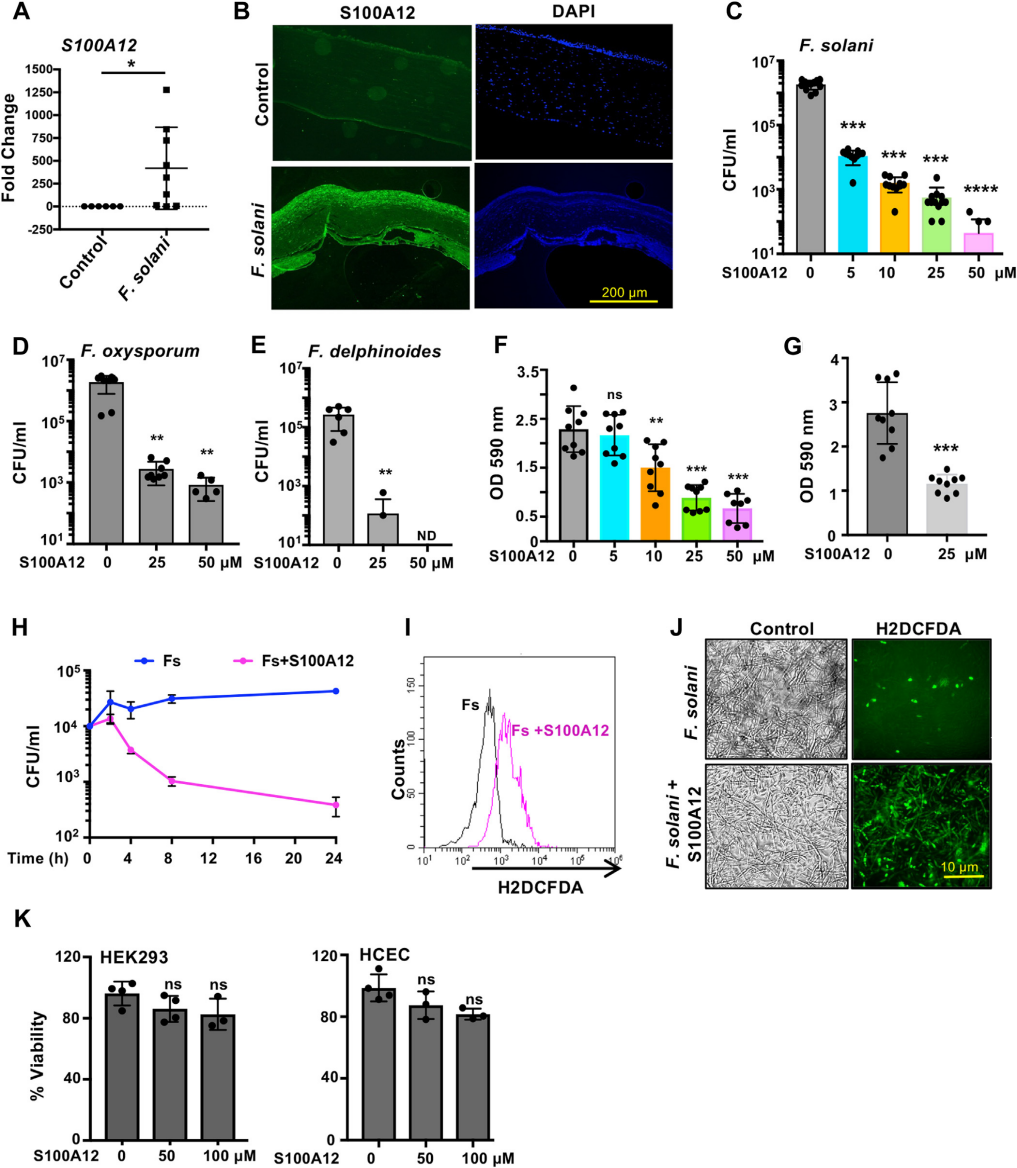
**S100A12 inhibits growth and biofilm formation of *Fusarium spp.*** S100A12 expression in corneal scrapings (n = 9) and corneal tissues (n = 4) was determined by quantitative PCR (*A*) and immunocytochemistry (*B*), respectively, in patients with fusarium keratitis. S100A12 inhibits growth of *Fusarium solani* (*C*), *Fusarium oxysporum* (*D*), and *Fusarium delphinoides* (*E*) as determined by colony-forming unit (c.f.u.). S100A12 prevents biofilm formation (*F*) and acts against preformed biofilms (*G*). The inhibition kinetics of S100A12 (25 μM) against *F. solani* was determined by c.f.u. *H,* ROS generation in *F. solani* by S100A12 (25 μM) was determined by flow cytometry (*I*) and fluorescence microscopy (*J*). No cytotoxic effect of S100A12 (50 and 100 μM) was observed in HEK293 and HCEC (*K*). All experiments were done at least three times in duplicates or triplicates, and data from all experiments are represented in the graph. (∗denotes <0.05, ∗∗denotes <0.005, and ∗∗∗denotes <0.0005, ns denotes not significant, ND denotes not determined). HCEC, human corneal epithelial cell; HEK293, human embryonic kidney cell; ROS, reactive oxygen species.

While screening for expression of host defense peptides in *Fusarium solani* keratitis patients, we found significantly increased expression of both mRNA (Fig. 1*A*) and protein (Fig. 1*B*) of S100A12 in the corneal scrapings and corneal tissues, respectively, compared with control cadaveric corneas. The S100A12 expression in human corneal epithelial cells (HCECs) in response to *Fusarium* infection over time was further confirmed by immunoblot assay along with its constitutive expression (Fig. S1*A*). S100A12 exhibited significant antifungal property against *F. solani* with reduction in fungal growth of more than 95% at 5 μM and more than 99% at 10, 25 μM, and higher concentrations of S100A12 (Fig. 1*C*). Significant reduction in colony-forming unit (c.f.u.) was also observed in S100A12 treated clinical isolates of *Fusarium oxysporum* (Fig. 1*D*) and *Fusarium delphinoides* (Fig. 1*E*) known to cause keratitis. *Fusarium* *spp.* has the ability to form highly structured biofilms that are more resistant to antifungal agents than planktonic cells (7). S100A12 at 10, 25, or 50 μM could significantly inhibit between 50% and 80% biofilm formation by *F. solani* (Fig. 1*F*). In addition, we checked if S100A12 could abolish preformed biofilms and found significant reduction (*p* < 0.0001) of preformed biofilms made by *F. solani* on treatment with S100A12 (25 μM) compared with the control (Fig. 1*G*). We next checked the kinetics for growth inhibition of *F. solani* by S100A12 and found differences in c.f.u. within 2 h of peptide incubation, which decreased significantly (85.87%) by 4 h (Fig. 1*H*) and reduced further by 99% at 24 h. Next, we determined if S100A12 could trigger generation of reactive oxygen species (ROS) in *Fusarium* and found significant increase in ROS within 2 h by flow cytometry (Fig. 1*I*) and fluorescence microscopy (Fig. 1*J*) in *Fusarium* *spp.* incubated with S100A12 (25 μM) compared with untreated *Fusarium*. However, there was no distinct ROS generation in HCEC incubated for 2 h in the presence or absence of S100A12 (25 μM) (Fig. S1*B*). In contrast to its action against fungal spores, S100A12 did not show any substantial cytotoxicity toward mammalian cells like HCEC or human embryonic kidney cells (Fig. 1*K*) even at higher concentrations of 50 μM or 100 μM, which implies that S100A12 exhibits selectivity toward fungal membrane but not to mammalian cell membrane.

### S100A12 binds to fungal membrane, causes membrane disruption in *Fusariumspp.* and reduces fungal burden in murine model of corneal infection

**Figure 2:**
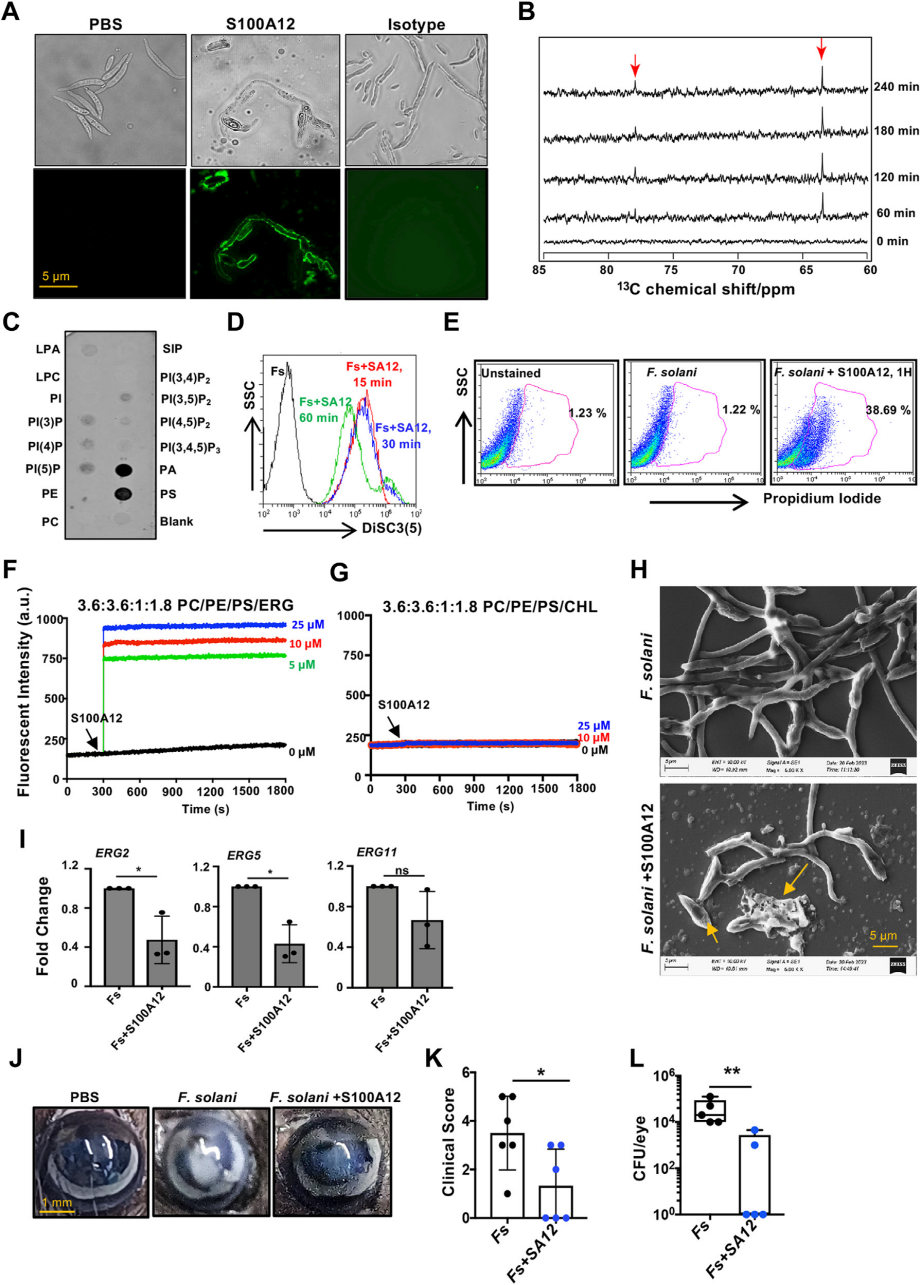
**S100A12 directly binds and damages membrane of*****Fusarium spp.**A,* binding of S100A12 to membrane of *Fusarium solani* was determined by confocal microscopy. *B,* interaction of S100A12 with fungal membrane to release secondary metabolites was determined using ^1^H-coupled ^13^C NMR spectroscopy. *C,* phospholipid binding of S100A12 (25 μM) to the PIP strip. *D,* S100A12 (25 μM) caused disruption of membrane potential in *F. solani* and was detected by 3,3′-diprophylthiadicarbocyanine iodide using flow cytometry. *E,* the membrane damage was detected in S100A12 (25 μM)-treated *F. solani* by the uptake of PI by flow cytometry. *F,* calcein dye leakage was observed in artificial liposomes made of PC/PE/PS/ERG on treatment with S100A12 (25 μM). *G,* no dye leakage was observed in liposomes made of PC/PE/PS/CHL that mimic mammalian membrane. *H,* scanning electron microscope images of *F. solani* incubated with S100A12 (25 μM) for 6 h. *I,* the gene expression of *ERG2*, *ERG5,* and *ERG11* was determined by quantitative PCR in *F. solani* incubated with or without S100A12 (25 μM). All experiments were repeated at least twice in duplicates, and data are representation from a single experiment. C57BL/6 mice (n = 6) were infected with *F. solani* and topically treated with S100A12 (100 μM) at 0 and 6 h postinfection. Mice were euthanized 48 h postinfection, and representative images of corneal opacification (*J*) and their clinical score (*K*) were recorded. *L,* colony-forming unit was measured from whole-eye homogenate 48 h postinfection, data points represent individual infected corneas. (∗denotes <0.05, ∗∗denotes <0.005, and ns denotes not significant). CHL, cholesterol; ERG, ergosterol; PC, phosphatidylcholine; PE, phosphatidylethanolamine; PI, propidium iodide; PS, phosphatidylserine.

Next, in an attempt to elucidate the mode of action of S100A12 on *Fusarium* *spp.*, we examined if S100A12 binds to the fungal membrane and causes membrane disruption. As revealed by confocal laser microscopy, S100A12 binds directly to fungal membrane of live *Fusarium* within 2 h of incubation (Fig. 2*A*). The changes in fungal membrane were also observed by using a series of one-dimensional ^1^H-coupled ^13^C NMR spectra. *Fusarium* grown on ^13^C-labeled glucose media released ^13^C-labeled metabolites on lysis by S100A12, which was observed as new peaks on the NMR spectra within 1 h (Fig. 2*B*). We next determined the specific membrane phospholipid that S100A12 might bind in *F. solani* by protein–lipid overlay assay. S100A12 was found to strongly bind to phosphatidic acid (PA), an anionic phospholipid, and phosphatidylserine (PS), and weakly to multiple phosphoinositides (Fig. 2*C*). We further checked the binding of S100A12 to cholesterol (CHL) in similar manner and did not find any specific binding (data not shown). S100A12 (25 μM) also caused disruption of membrane potential in *F. solani* within 15 min that was consistent after 60 min of exposure (Fig. 2*D*) by using fluorescent voltage reporter DiSC_3_ (5). Using nucleic acid staining dye propidium iodide (PI) that permeates fungal cell membrane when compromised, the PI influx was detected in live *F. solani* within 1 h of incubation with S100A12 (25 μM) by flow cytometry (Fig. 2*E*). This was further confirmed by calcein leakage assay using artificial liposomes that mimicked fungal or mammalian model systems. It is noteworthy to mention that the major difference between mammalian and fungal membrane lies in the difference of sterol moiety composition; ergosterol (ERG) is present in fungal membrane, whereas CHL is present in mammalian membrane (8). Calcein dye encapsulated large unilamellar vesicles (LUVs) composed of phosphatidylcholine (PC)/phosphatidylethanolamine (PE)/PS/ERG (3.6:3.6:1:1.8) served as a fungal membrane model mimics in this study (9). S100A12 interacted with LUVs, perturbing them, and resulting in the release of calcein in the solution, which was detected with an increase in fluorescence intensity after treatment with increasing concentration of S100A12 (Fig. 2*F*). In contrast, no leakage of dye was noted in LUVs made of PC/PE/PS/CHL (3.6:3.6:1:1.8) (Fig. 2*G*). Interestingly, S100A12 did not show any dye leakage effect in 6:4 PC/CHL LUV (Fig. S2*A*), widely considered as model mammalian membrane system (10). Evidently, no membrane disruption causing dye leakage was also observed in liposomes devoid of any sterol moieties (Fig. S2*B*). In addition, no significant uptake of PI influx was also observed in corneal epithelial cells incubated with S100A12 (25 μM) for 2 h (Fig. S2*C*). The effect of S100A12 on membrane morphology of *F. solani* was further examined by scanning electron microscopy. Significant damage on the surface of the membrane causing membrane ruptures along with membrane debris was observed in *F. solani* treated with S100A12 (25 μM) for 6 h, compared with the untreated fungus (Fig. 2*H*). We next checked the effect of S100A12 on expression of genes involved in synthesis of ERG in fungal cell membrane. S100A12 (25 μM) significantly reduced the gene expression of *erg2* and *erg5* (Fig. 2*I*) involved in last steps of ERG biosynthesis. S100A12 also reduced the expression of *erg11*, a common target of available azole antifungals. Finally, we evaluated the therapeutic effect of S100A12 in a well-established murine model of FK (11, 12). Increased corneal disease marked by corneal opacification was detected in mice (n = 6) infected with *F. solani* (Fig. 2*J*ii), but significantly, less opacification was observed in infected mice treated with S100A12 (100 μM) (n = 6) (Fig. 2*J*iii). Significant differences were noted in the clinical scores of corneal opacities between infected mice and mice treated with S100A12 (Fig. 2*K*) indicating reduction in disease development. Consistent with these data, c.f.u. per eye obtained were significantly less in S100A12 treated mice compared with mice infected with *F. solani* (Fig. 2*L*). These data clearly indicate that S100A12 is effective and selective in inhibiting the growth of *Fusarium* *spp.*

## Discussion

The drawbacks of the current antifungals including toxicity toward mammalian cells make it necessary to develop new therapeutics to treat fungal infections. Although antibacterial property of S100A12 has been explored earlier, studies on antifungal property of S100A12 remained elusive. In the current study, we demonstrated antifungal property of S100A12 against filamentous *Fusarium* *spp.,* a major human pathogen, thus widening the scope of potential application of the peptide. There is a scarcity of reports of host defense peptides effective against *Fusarium* *spp.* in general.

There are many evidences indicating the presence of S100A12 in several inflammatory diseases, like rheumatoid arthritis and psoriasis (13), as well as in infections (4, 5, 14). We found significant increased expression of both mRNA and protein levels of S100A12 in patients with fusarium keratitis. Because of its increased abundance in fusarium-infected corneal tissues, we checked the antifungal property of S100A12 and found that it significantly inhibits growth and viability of *F. solani* and few other *Fusarium* *spp.* Although host defense peptides are present endogenously, often they are expressed in suboptimal concentration not enough to combat as acute infection sets in. However, they are effective against the pathogens *in vitro* and holds the potential to be developed as therapeutics (15). In addition, S100A12 was not toxic to mammalian cells even at higher concentrations and therefore appears to be predominantly specific in its action toward *Fusarium* *spp.* The formation of fungal biofilm contributes to its increased resistance toward antifungals and makes treatment against fungal infections extremely challenging. S100A12 not only inhibited biofilm formation by *F. solani* but also significantly reduced the preformed biofilms. AMPs like ToAP2 and EC1-17KV have been earlier found to inhibit biofilm formation by *Candida albicans* (16, 17). In the current study, we found S100A12 to induce ROS generation in *F. solani*, but no ROS was triggered in human corneal epithelial cells*.* In the mechanistic studies using S100A12, we found that S100A12 binds directly to fungal cell membrane and causes membrane permeabilization and disruption of membrane potential early on leading to inhibition of filament formation. This result is consistent with several other studies that suggest action of AMPs is by targeting and disrupting the cell membrane of fungus or bacteria (17, 18). However, spores temporarily exposed to S100A12 for 2 h were able to germinate later (data not shown). It was seen from the kinetics study that reduction of fungal viability occurred within 2 h, which coincided with the time points of membrane permeabilization and ROS generation; however, whether these are directly responsible for inhibition of fungal growth or exert additional secondary effects need further evaluation. Phospholipids are the main constituents of the fungal membrane and influence cell growth and virulence (19). Interestingly, we found that S100A12 strongly bound to anionic PS and PA along with other phosphatidylinositol diphosphates. Both PA and PS are involved in the early steps of phospholipid biosynthesis, and disruption of membrane potential early on, along with ROS generation, might have resulted in the externalization of PS that are present in the inner leaflet of membrane. However, the peptide did not show any significant binding to PE or PC, which are also abundant in fungal membrane. The membrane disruption ability of S100A12 was also tested using liposomes mimicking the fungal and mammalian model membrane. The effect of S100A12 on LUV composed of fungal (PC/PE/PS/ERG) or mammalian (PC/CHL) membranes was examined. No leakage of calcein was observed in the case of LUV made up of PC/PE/PS/CHL validating that the peptide is selective and targets ERG containing membrane and not one with CHL. In other words, S100A12 specifically causes disruption of fungal membrane and not of mammalian membrane. This finding is consistent with the fact that there was no difference in uptake of PI in HCEC in the presence or the absence of S100A12 indicating the lack of membrane damage. It has also been reported with other peptides that the presence of CHL in cell membranes classically inhibits the binding and lytic activity of AMPs (20). The data from scanning electron microscope also clearly showed that S100A12 causes membrane rupture and inhibited hyphal growth of the spores to a large extent. Further studies are needed to understand the detailed mechanism by which membrane permeabilization is induced by S100A12 and if any additional methods are involved. We also showed S100A12 to be effective in inhibiting *F. solani* corneal infections *in vivo* with significant reduction of corneal opacity and fungal burden in infected corneas treated with S100A12 compared with untreated ones. Since mice do not encode for S100A12 (21), reduction in fungal burden in mice topically administered with S100A12 demonstrated its direct protective effect in corneal infection. Clark *et al.* (22) have also earlier shown S100A8/S100A9 to be effective *in vivo* in regulating hyphal growth in *Aspergillus fumigatus* corneal infections. Although amphotericin B, the commonly used antifungal, is effective against *Fusarium* *spp.*, it is known to be cytotoxic against mammalian cells (23). In contrast, S100A12 not only was effective in inhibiting growth and biofilm formation of *Fusarium* but also was not cytotoxic to mammalian cells and did not induce ROS generation or membrane damage in these cells.

In summary, this study exhibits the potentials of an endogenous host peptide, S100A12, that not only inhibits growth or biofilm formation but also causes membrane permeabilization by direct binding to phospholipids and induces ROS in *Fusarium* *spp.* It also demonstrates therapeutic potentials of S100A12 against *F. solani*-induced corneal infections *in vivo*. Therefore, the data provide a *proof of principle* that S100A12 may serve as a possible novel intervention in treating *Fusarium* infections.

## Experimental procedures

### Collection of corneal scrapings

Corneal scrapings were collected from patients with FK after obtaining written informed consents, and the protocol was approved by the Institutional Review Board of Hyderabad Eye Research Foundation, India, and research followed tenets of the Declaration of Helsinki. The clinical characteristics of patients are shown in Table S1. Cadaveric corneas unsuitable for transplant were used as controls and obtained from Ramayamma International Eye Bank, LVPEI, India. Archived corneal tissue sections of fusarium keratitis patients surgically removed during penetrating keratoplasty were obtained from the Department of Pathology, LVPEI.

### Culture of *Fusariumspp.*

The clinical isolates of *F. solani*, *F. oxysporum*, and *F. delphinoides* were obtained from Jhaveri Microbiology Centre, LVPEI. The isolates were grown at 25 °C in Sabouraud dextrose broth (Himedia) for 48 h. Conidia (spores) were harvested by filtering the culture through sterile cotton gauze to remove hyphae and centrifuged at 8000 rpm for 15 min to pellet the conidia and resuspended in sterile 1× PBS as described before (24).

### Recombinant S100A12

S100A12 was purified as described before (25). Protein concentration was determined by monitoring the absorbance at 280 nm using an extinction coefficient of 2980 M^−1^ cm^−1^ (4).

### Inhibition of growth of *Fusariumspp.* by S100A12

*Fusariumspp.* (10^4^ conidia) was incubated with various concentrations of S100A12 in Sabouraud dextrose broth for 24 h at 25 °C. The cultures were then plated on potato dextrose agar (PDA; Himedia) plates in serial dilutions and incubated further for 24 h at 25 °C. The viability of the fungus was determined by quantifying the c.f.u. on the plates.

### Time-kill kinetics of S100A12 against *Fusarium*

Time-kill kinetics of S100A12 against *F. solani* was performed according to the standard techniques with few modifications (26). *F. solani* (10^4^ conidia) was incubated with S100A12 (25 μM) at 25 °C for different time intervals (0, 2, 4, 8, and 24 h). To quantify the fungal growth, cultures were serially diluted, plated on PDA plates, and incubated further at 25 °C for 24 h. The kinetics of S100A12 activity was shown by plotting total c.f.u. against time. The culture medium was used as negative control.

### Biofilm assay

The effect of S100A12 against biofilm formed by *Fusarium* *spp.* was determined as described earlier (27). In brief, *F. solani* (10^4^ conidia) was incubated with different concentrations of S100A12 for 24 h at 37 °C. In a separate experiment, the biofilm was first formed for 24 h followed by further incubation with S100A12 (25 μM) for 24 h. The supernatants were removed, washed with 1× PBS, and biofilms were fixed by 95% methanol for 15 min, followed by staining with 0.5% of crystal violet (Sigma–Aldrich). The wells were rinsed, dye was dissolved in 30% acetic acid, and absorbance was measured at 570 nm by SpectraMax M3 Reader (Molecular Devices).

### Immunohistochemistry

Tissue sections (5 μm) of paraffin-embedded corneas diagnosed with *F. solani* keratitis were deparaffinized and stained with anti-S100A12 antibody (1:100 dilution; Novus Biologicals) as described earlier (6), counterstained with 4′,6-diamidino-2-phenylindole (Abcam) and observed under fluorescent microscope (Olympus IX73; Zeiss) using 20× objective and imaged using Olympus DP71 camera. Cadaveric corneas unsuitable for transplantation were used as control.

### Cytotoxicity assay

Cytotoxicity in human embryonic kidney 293 and HCEC 10.014 pRSV-T (28) was determined using 3-[4,5-dimethylthiazol-2-yl]-2,5 diphenyl tetrazolium bromide (Sigma–Aldrich) cleavage assay as described earlier (29). The percent viability was calculated as (absorbance value of experiment well)/(absorbance value of control well) × 100.

### ROS measurement

The intracellular ROS in *F. solani* was measured using 2′,7′-dichlorofluorescin diacetate (Invitrogen) as described (30). *F. solani* (10^6^ conidia/ml) were incubated with 2′,7′-dichlorofluorescin diacetate in the presence or the absence of S100A12 (25 μM) for 2 h, washed with 1× PBS, and checked by fluorescence microscope (Axio Vert.A1; Zeiss) using 20× objective or analyzed by flow cytometry (Beckman Coulter). The mean fluorescence intensity was determined from two separate experiments. ROS generation in HCEC was also determined in a similar manner.

### Scanning electron microscopy

*F. solani* (10^4^ conidia) were grown on glass cover slips in the presence or the absence of S100A12 (25 μM) for 6 h, washed, and fixed with 4% paraformaldehyde. After fixation, cover slips were washed, dehydrated with graded ethanol (10–100%), and air dried overnight. The cover slips were then sputtered with gold palladium for 75 s with high vacuum evaporator and visualized using a scanning electron microscope (Zeiss-Model EVO 18; Carl Zeiss) at a magnification of 5000×.

### Membrane depolarization

*F. solani* (10^4^ conidia) were left untreated or incubated with S100A12 (25 μM) for different time points, washed with 1× PBS, and incubated with 250 nM of 3,3′-dipropylthiadicarbocyanine iodide (DiSc_3_-5; Sigma–Aldrich) for 30 min in dark followed by addition of 100 mM KCl as described earlier (31). The membrane potential was determined by flow cytometry (Beckman Coulter).

### PI uptake and PI staining assay

*F. solani* (10^6^ conidia) was incubated in the absence or the presence of S100A12 (25 μM) for 1 h, washed with 1× PBS, and further incubated with PI (1 mg/ml) for 15 min. The uptake of PI was analyzed by flow cytometry (Beckman Coulter). The PI uptake was also checked in HCEC incubated with S100A12 (25 μM) for 2 h following the same procedure using fluorescent microscope (Axio Vert.A1; Zeiss) at 20×.

### Binding of S100A12 to fungal cell wall

*Fusariumspp.* (10^4^ conidia) were incubated with 25 μM of S100A12 at 25 °C for 1 h. The conidia were fixed using 4% paraformaldehyde followed by incubation with anti-S100A12 antibody (1:100 dilution; Novus Biologicals) for 45 min, washed, and further incubated with goat anti-rabbit Alexa Fluor 488 secondary antibody (1:1000 dilution; Invitrogen) for 1 h. The conidia were further washed and imaged using confocal microscope (LSM 880; Zeiss).

### ^1^H-coupled ^13^C NMR experiment

*Fusariumspp.*were grown in media containing yeast extract, peptone, and ^13^C-labeled glucose for 48 h under 120 rpm, harvested, washed thoroughly, and resuspended in 10 mM phosphate buffer (pH 7.4). About 10^7^ spores/ml was taken in NMR tube, and all NMR experiments were performed at 298 K on a Bruker Avance III 700 MHz NMR spectrometer. About 10% deuterated water and 3-(trimethylsilyl)-2,2,3,3-tetradeuteropropionic acid were used for locking and internal standard (0.00 ppm), respectively. First, a free 1D ^1^H NMR spectrum of the free cell was recorded followed by a series of 1D ^1^H (water suppression using excitation sculpting) and ^1^H-coupled ^13^C-NMR (Bruker pulse program: zggd30) spectra recorded after addition of 0.5 mM S100A12, and new peaks were observed in the 1D ^13^C NMR spectra with time.

### Liposome preparation and calcein dye leakage assay

The stock solutions of 1,2-dioleoyl-*sn*-glycero-phosphocholine (DOPC), 1,2-dioleoyl-*sn*-glycero-3-phosphoethanolamine (DOPE), and 1,2-dioleoyl-*sn*-glycero-3-phospho-l-serine (DOPS) (Avanti Polar Lipids, Inc) and ERG and CHL (Sigma–Aldrich) were prepared in chloroform (25 mg/ml). Preparation of fungal model membrane mimics comprising 3.6:3.6:1:1.8 DOPC/DOPE/DOPS/ERG and mammalian model membrane comprising 6:4 POPC/CHL followed by dye leakage experiments was done as published earlier (10, 32). For direct investigation on the role of sterol moieties, model membrane mimics composed of 3.6:3.6:1:1.8 DOPC/DOPE/DOPS/CHL as well as 3.6:3.6:1 DOPC/DOPE/DOPS were used as control. The detailed dye leakage protocol has been provided in the supporting information.

### S100A12–phospholipid binding assay

S100A12–phospholipid interactions were determined using PIP Strips (Molecular Probes) as described previously (13) with minor modifications. The binding of S100A12 (25 μM) to the phospholipids on strip was detected using anti-S100A12 antibody (1:100 dilution; Novus Biologicals) followed by incubation with IRDye-680 secondary antibody (1:6000 dilution; LI-COR Biotechnology) and were developed on Odyssey CLx Imaging System (LI-COR Biotechnology).

### RNA extraction, complementary DNA synthesis, and quantitative PCR

*F. solani* (10^7^ spores) were treated or not with S100A12 (25 μM) for 6 h. For total RNA isolation, mycelia were incubated with 1% of Triton X-100 for 30 min followed by extraction with Trizol (Invitrogen), and purified by RNAeasy kit (Qiagen) according to the kit protocol. RNA was quantified using NanoDrop 2000c Spectrophotometer (Thermo Scientific) and reverse transcribed using Verso complementary DNA synthesis kit (Thermo Scientific) according to kit protocol. Quantitative PCR was performed on ABI PRISM 7000HT Sequence Detection System (Applied Biosystems) using SYBR Green PCR Master Mix (Thermo Fisher). The primer sequences are listed in Table S2. Relative quantities of mRNA expression of respective genes were normalized using the 2^−ΔΔct^ method using 18s rRNA as the housekeeping gene.

### Murine model of keratitis

C57BL/6 mice (6–8 weeks of age) were anesthetized by intraperitoneal injection using ketamine and xylazine as stated before (33). The corneal epithelium was abraded with three parallel vertical and horizontal scratches with a 26-gauge needle, and *F. solani* (10^7^ conidia/2.5 μl) were added in one eye, whereas fellow eye received 1× PBS (n = 6). In another group (n = 6), mice were infected with *F. solani*, and S100A12 (100 μM) was topically added to corneas at 0 and 6 h postinfection. Mice were euthanized and examined under a stereomicroscope for corneal opacification or ulceration 48 h postinfection and photographed using a point shoot camera. Clinical scores for opacity were determined in blinded fashion according to the scale earlier reported (34), eyes were enucleated and homogenized using a tissue homogenizer (Genetix Biotech) in 1× PBS, and serial dilutions of homogenates were plated on PDA plates for determining c.f.u. All animals were housed in pathogen-free conditions in microisolator cages and were treated in accordance with the guidelines provided in the ARVO Statement for the Use of Animals in Ophthalmic and Vision research. The study was approved by the Institutional Animal Ethics Committee (EAF/SR/15/2023), CDFD, India.

### Statistical analysis

Bar graphs denote mean, and error bars denote SD. All data were statistically analyzed, for comparisons between control and treated, two-tailed, unpaired *t* tests were performed. Mann–Whitney test was used for animal experiments. All statistical analyses were done using Prism 7 (GraphPad Software, Inc). Results were considered statistically significant when *p* < 0.05.

## Data availability

All data described in this study are contained within the main article and supporting information.

## Supporting information

This article contains supporting information (10, 32).

## Conflict of interest

The authors declare that they have no conflicts of interest with the contents of this article.
