# Supplementary material for: Host antimicrobial peptide S100A12 disrupts the fungal membrane by direct binding and inhibits growth and biofilm formation of Fusarium species
Source: J Biol Chem. 2024 Jan 30;300(3):105701. doi: 10.1016/j.jbc.2024.105701 (PMC10891332; doi:10.1016/j.jbc.2024.105701)
Supplement: Supporting information [file mmc1.pdf]

Supporting Information

Host antimicrobial peptide S100A12 disrupts the fungal membrane by direct binding and inhibits growth and biofilm formation of *Fusarium species*

Sanhita Roy,[1,*] Bharathi Bhogapurapu,[1] Sreyanki Chandra,[1] Karishma Biswas,[3] Priyasha Mishra,[1,2] Abhijit Ghosh[1], Anirban Bhunia[3]

1. *Prof. Brien Holden Eye Research Centre, LV Prasad Eye Institute, Hyderabad 500034, India*
2. *Manipal Academy of Higher Education, Manipal, India*
3. *Department of Chemical Sciences, Bose Institute, Unified Academic Campus, Sector V, EN 80, Kolkata 700091, India*

[*]Corresponding author

Sanhita Roy, PhD

Prof. Brien Holden Eye Research Centre,

LV Prasad Eye Institute

Hyderabad-500034, India

Email: sanhita@lvpei.org

Telephone: +91-40-68102529; Fax: +91-40-68102535

Running Title: *S100A12 inhibits Fusarium spp.*

Supplementary experimental procedures

Western Blot

Human corneal epithelial cells (HCEC) were infected with *F. solani* at multiplicity of infection (MOI) of 1:100 (cells:bacteria) for 4 and 6 h and expression levels of endogenous S100A12 determined by western blot. Briefly, postinfection cells were washed with 1X PBS and lysed with 1X lysis buffer (CST, Danver, MA) and total protein was determined by BCA assay (Thermo Scientific, CA). Total protein was separated using 15% SDS-PAGE, transferred to nitrocellulose membrane and stained with Ponceau S (Sigma-Aldrich, St. Louis, MO) to determine the total protein transferred. The lysates of *E. coli* BL21 transformed with S100A12 plasmid along with the untransformed bacterial lysates and purified protein were also loaded in the gel and transferred to the same membrane. The membrane was then probed with anti-S100A12 antibody (1:2000; Novus Biologicals, CO) and further incubated with IRDye-680 secondary antibody (1:6000; LI-COR Biotechnology, Lincoln, NE) and was developed on Odyssey CLx Imaging System (LI-COR Biotechnology, NE).

Calcein dye leakage assay

The detailed protocol for dye leakage has been published previously (1, 2). Briefly, lipid film was created after drying in a stream of nitrogen gas and lyophilizing overnight. The lipid film was then hydrated with 70 mM calcein dissolved in 10 mM Tris buffer (pH 7.4), followed by vigorous vortexing for 30 min and subjecting to five freeze-thaw cycles in liquid nitrogen and lukewarm water to obtain dye entrapped vesicles. The vesicles were then passed through a 100 nm polycarbonate membrane filter stacked in a mini extruder (Avanti Polar Lipids, Alabaster, AL) set up to obtain unilamellar vesicles (LUVs). The free calcein dye was removed by passing the extruded samples through a gel-filtration based hydrated Centrisep-Spin Column (Thermo Fisher Scientific, Waltham, MA) which was centrifuged at 3000 rpm for 2 min. Dye-encapsulated liposomes were suspended in an extra vesicular buffer containing 10 mM Tris buffer, 100 mM NaCl (pH 7.4). The calcein leakage caused by liposome disruption was analyzed by fluorescence emission at 519 nm by JASCO F-8500 fluorescence spectrophotometer (Easton, MD).

The slit width for both excitation and emission were kept at 5 nm. The vesicles were allowed to stabilize in buffer. After stabilization, an increasing concentration of S100A12 was added to the vesicles. The effect of each peptide concentration on the vesicles was measured for 30 mins respectively and the enhancement in the fluorescence intensity was recorded.

Supplementary figures

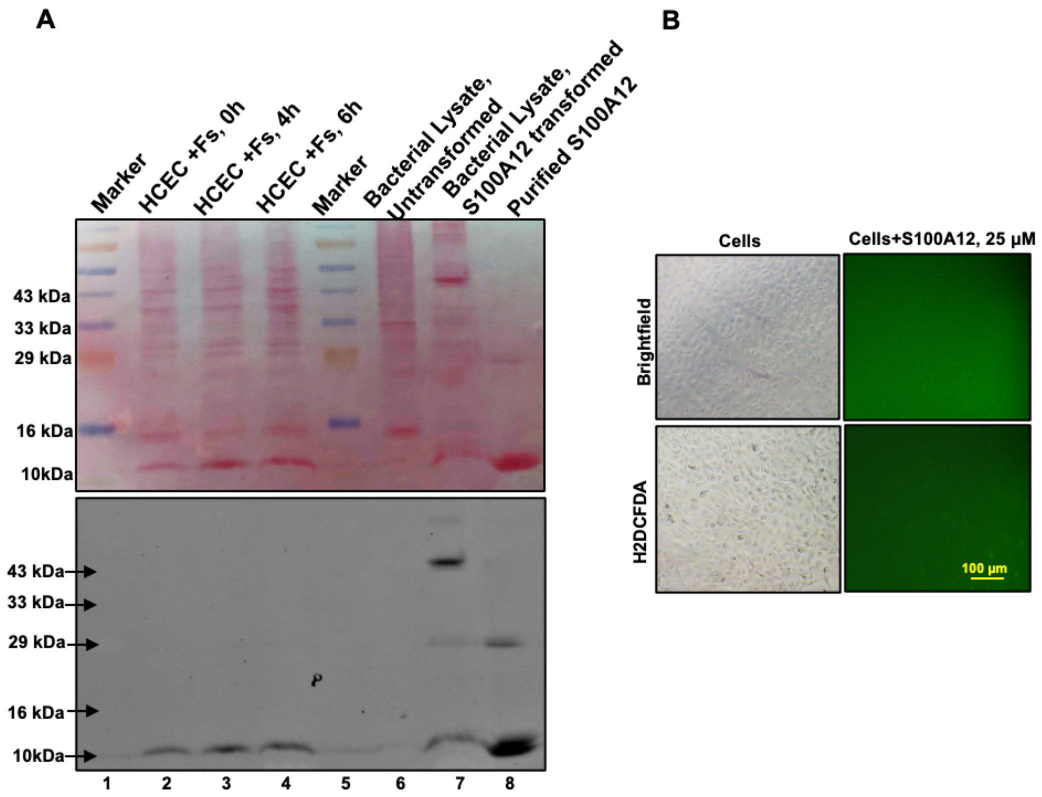

Figure S1. S100A12 expression in human corneal epithelial cells (HCEC) infected with *F. solani* at MOI 100 for 0, 4 and 6 h (lanes 2,3 and 4) and untransformed (lane 6) or transformed (lane 7) bacterial lysates and purified protein as detected by western blot analysis (lower panel). The ponceau stain (upper panel) of the membrane shows the total protein loaded in gel (A). The generation of reactive oxygen species (ROS) was determined in HCEC in presence or absence of S100A12 using H2DCFDA (B).

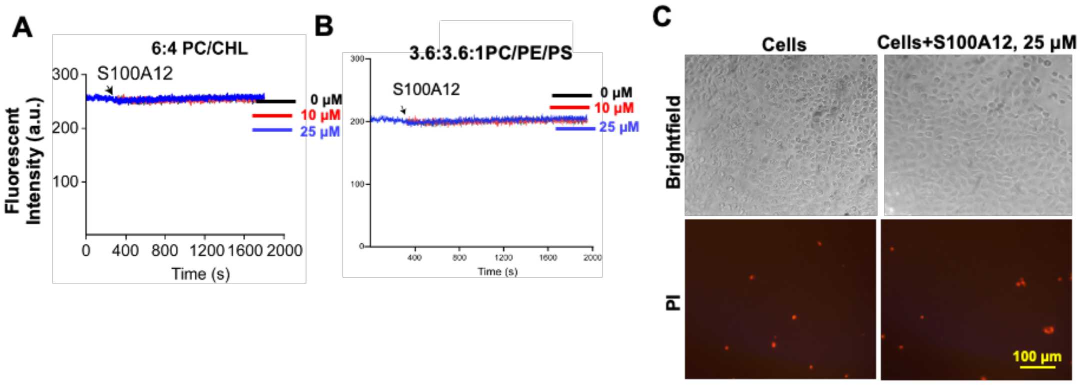

Figure S2. Calcein dye leakage assay was performed to check the membrane damage in artificial liposomes incubated with different concentration of S100A12. No leakage was observed in liposomes made of PC/CHL (A) and PC/PE/PS (B). No significant difference in uptake of propidium iodide was detected in HCEC in presence or absence of S100A12 for 2 h (C).

Table S1: Clinical characteristics of patients

**Characteristics**	***F. solani***
**Age**	29 to 80
Mean	50.44
(SEM)	(5.7)
**Sex**	
Male (%)	66
Female (%)	34
**Hypopyon**	
Yes (%)	33
No (%)	67
**Occupation**	
Agriculture/ Manual Labour (%)	77
Desk jobs (%)	11
Unspecified (%)	12
**Surgery**	
Yes (%)	45
No (%)	55

Table S2: Oligonucleotide sequences

**Gene**	**Primers (5-3')**
erg2	FWD: AGTCCAGCAAGCCCTCGTCA REV: TGCTCGGGGTTCTTCTCGCT
erg5	FWD: GAGCAGAAGAAGAGCGGAATGC REV: TAAAGTGAGGCAGGACGACCAC
erg11	FWD: CCTTTGGTGCCGGTAGACAT REV: CCCATCGAATAAACGCAGGC
18s rRNA	FWD: CGCCAGAGGACCCCTAAAC REV: ATCGATGCCAGAACCAAGAGA

References:

1. Pariary, R., Ghosh, B., Bednarikova, Z., Varnava, K. G., Ratha, B. N., Raha, S., Bhattacharyya, D., Gazova, Z., Sarojini, V., Mandal, A. K., and Bhunia, A. (2020) Targeted inhibition of amyloidogenesis using a non-toxic, serum stable strategically designed cyclic peptide with therapeutic implications. *Biochim Biophys Acta Proteins Proteom* **1868**, 140378.

2. Mohid, S. A., Biswas, K., Won, T., Mallela, L. S., Gucchait, A., Butzke, L., Sarkar, R., Barkham, T., Reif, B., Leipold, E., Roy, S., Misra, A. K., Lakshminarayanan, R., Lee, D., and Bhunia, A. (2022) Structural insights into the interaction of antifungal peptides and ergosterol containing fungal membrane. *Biochim Biophys Acta Biomembr* **1864**, 183996
